# Lipoprotein(a): from Causality to Treatment

**DOI:** 10.1007/s11883-024-01187-6

**Published:** 2024-01-22

**Authors:** Florian Kronenberg

**Affiliations:** Institute of Genetic Epidemiology, Medical University of Innsbruck, Schöpfstrasse 41, 6020 Innsbruck, Austria

**Keywords:** Lipoprotein(a), Epidemiology, Genetics, Cardiovascular disease, mRNA targeting therapy

## Abstract

**Purpose of Review:**

This paper reviews the evidence why lipoprotein(a) (Lp(a)) is a causal risk factor for cardiovascular disease and how high Lp(a) concentrations should be managed now and with an outlook to the future.

**Review Findings:**

No optimal and widely available animal models exist to study the causality of the association between Lp(a) and cardiovascular disease. This has been a major handicap for the entire field. However, genetic studies turned the page. Already in the early 1990s, the principle of Mendelian randomization studies was applied for the first time ever (even if they were not named so at that time). Genetic variants of the *LPA* gene such as the apolipoprotein(a) isoform size, the number and sum of kringle IV repeats and later single nucleotide polymorphisms are strongly associated with life-long exposure to high Lp(a) concentrations as well as cardiovascular outcomes. This evidence provided a basis for the development of specific Lp(a)-lowering drugs that are currently in clinical testing phase.

**Summary:**

Lp(a) is one of the most important genetically determined risk factors for cardiovascular disease. With the specific Lp(a)-lowering therapies, we might get tools to fight this common risk factor in case the outcome trials will be positive.

## Introduction

**Fig. 1:**
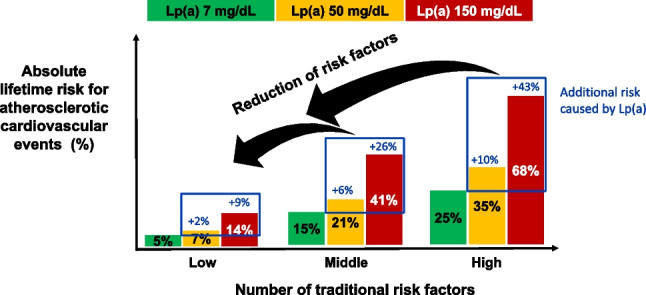
This figure is based on calculations for the Lp(a) Consensus statement of the European Atherosclerosis Society (presented as Fig. 6 therein [6••]) and presents the main message in a simplified form. The *y*-axis shows the estimated absolute lifetime risk for major atherosclerotic cardiovascular events (ASCVD) among 415,274 participants of European ancestry in the UK Biobank. Participants are divided into categories of baseline estimated lifetime risk of 5% which equals no or a low number of traditional risk factors, 15% (medium number of risk factors), and 25% (high number of risk factors), respectively, calculated using the Joint British Societies (JBS3) Lifetime Risk Estimating algorithm (based on traditional risk factors age, sex, blood cholesterol, blood pressure, smoking, diabetes, family history of heart attacks in early life, and BMI). For each of these three baseline risk categories, the additional risk attributable to increasing Lp(a) concentrations of 50 mg/dL (*yellow bars*) or 150 mg/dL (red bars) measured at baseline compared to those with the median Lp(a) concentration of 7 mg/dL (green bars) is calculated and added to the baseline risk to provide the global absolute risk. This incremental increase in risk caused by higher Lp(a) concentrations of 50 mg/dL and 150 mg/dL was estimated by adding Lp(a) as an independent exposure to the JBS3 risk estimating algorithm. For example, for a person with a baseline risk of 25% and an Lp(a) concentration of 50 mg/dL, the absolute risk of a major cardiovascular event increases by 10% from 25 to 35% (versus a person with an Lp(a) of 7 mg/dL). In case of an Lp(a) concentration if 150 mg/dL the risk increases by 43% from 25 to 68%. The reduction of modifiable traditional risk factors is therefore the ultimate goal in case of elevated Lp(a) concentrations to decrease the global risk of a given person

After Kåre Berg first described Lp(a) in 1963 [1], it took 10 years before the first observations were published by Dahlén and colleagues that high Lp(a) concentrations might be linked to cardiovascular disease [2, 3]. Further, approximately 10 years later, Kostner et al. introduced for the first time the 30 mg/dL and 50 mg/dL thresholds by describing that patients with myocardial infarction more frequently had Lp(a) concentrations above these two thresholds compared to controls [4]. Later, this 50 mg/dL threshold was recommended by the first Lp(a) European Atherosclerosis Society (EAS) consensus statement in 2010 and is equivalent to the 80th percentile of Lp(a) concentrations in a random general population from Denmark [5]. Meanwhile, we know that there is not really a threshold but there is a continuous relationship between Lp(a) concentrations and cardiovascular risk. That means, the higher the Lp(a) concentration, the higher the cardiovascular risk. This relationship was not only observed in White populations but also Black or Asian populations [6••, 7, 8]. Nevertheless, from a clinical point of view, thresholds are demanded. Therefore, the most recent EAS consensus statement from 2022 proposed that Lp(a) concentrations up to 30 mg/dL are not associated with a clinically meaningful risk increase and that concentrations above 50 mg/dL are associated with a clinically relevant risk increase and with a grey zone between 30 and 50 mg/dL [6••]. When looking at the relative risk increase in relation to Lp(a) concentrations, we can observe a continuous risk increase of 1.22-fold, 1.40-fold, 1.65-fold, 1.95-fold, and 2.72-fold at concentrations of 30 mg/dL, 50 mg/dL, 75 mg/dL, 100 mg/dL, and 150 mg/dL, respectively, when compared to those with median Lp(a) concentrations of 7 mg/dL. However, even more important is the absolute risk increase and this depends on the global risk which considers besides Lp(a) concentrations also the risk derived from traditional risk factors such as age, sex, blood cholesterol, blood pressure, smoking, diabetes, family history of heart attacks in early life, and BMI. Figure 1 clearly shows that even high concentrations of Lp(a) are not necessarily associated with an increased risk for cardiovascular disease. Two persons with the same high Lp(a) concentration of 150 mg/dL have an absolute risk of roughly 14% or 68% in case they have a baseline risk of 5% (corresponds to having no or a very low number of traditional risk factors) and 25% (very large number of traditional risk factors), respectively [6••, 9•].

## Genetic Variability Controls Lp(a) Concentrations

**Fig. 2:**
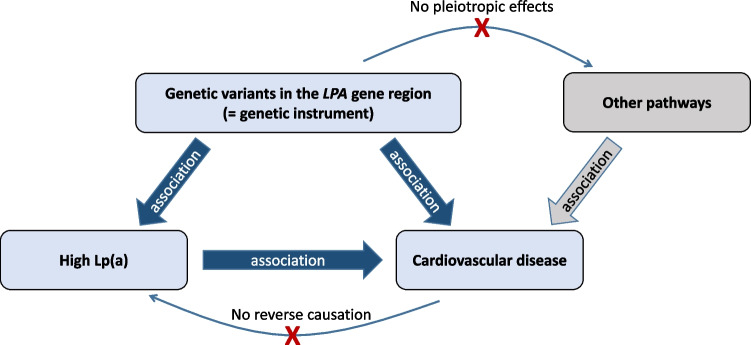
Schematic illustration of a Mendelian randomization approach using the example of lipoprotein(a) [Lp(a)]. Observational studies showed a significant association of high Lp(a) concentrations with cardiovascular disease. Genetic variants which show a strong association with high Lp(a) concentrations are used as genetic instrument (e.g., small apo(a) isoforms, low number of K-IV repeats, certain SNPs associated with high Lp(a) concentrations). When these instruments show also an association with cardiovascular disease, a causal association between Lp(a) concentrations and cardiovascular disease is strongly supported and a reverse causation might be excluded. Pleiotropy has to be excluded in the sense that the genetic variant affects the outcome only via the biomarker and not via other independent pathways. (Reproduced from [10], https://www.sciencedirect.com/science/article/pii/S1043661823001998?via%3Dihub; Creative Commons user license; https://creativecommons.org/licenses/by/4.0/)

During the early 1990s, there was a major discussion whether Lp(a) is a causal risk factor for cardiovascular complications or whether an elevated Lp(a) level only indicates that a cardiovascular disease might be present in a particular person. These two possibilities have very different consequences. In case Lp(a) is a causal risk factor for cardiovascular disease, efforts to lower Lp(a) therapeutically might be the next logical step. In case Lp(a) indicates only that a patient might have cardiovascular disease without a causal involvement of Lp(a) in disease development, Lp(a) might be “only” a diagnostic marker as it is the case for troponins which “mark” a damage of heart muscle cells. In case Lp(a) is only a marker, a therapeutic lowering would not have any consequences on disease development or progression since it reflects only a “reverse causation” (Fig. 2). The results from various case–control and prospective studies in the early 1990-ies were contrasting as discussed recently [11, 12••]. The pioneering work of Gerd Utermann and colleagues brought a turning point. They discovered in 1987 a size polymorphism of apolipoprotein(a) [apo(a)], which is apolipoprotein that characterizes the Lp(a) particles. This size polymorphism became the key for understanding the genetics of Lp(a) with apo(a) protein sizes ranging from 300 to > 800 kDa [13]. The later molecular characterization of *LPA*—the gene encoding apo(a)—showed that the protein size polymorphism is caused by a varying number of kringle-IV (KIV) repeats in the *LPA* gene [14–16]. Each of those KIV repeats has a size of 5.6 kb, and the repetitive structure of up to more than 30 apo(a) DNA size fragments results in a copy number variation. The size variation of DNA fragments corresponds to the size heterogeneity of protein isoforms in plasma and both co-segregate in families [15, 16].

Of similar importance was the observation that there exists an inverse correlation between apo(a) isoform size and Lp(a) concentrations [13]. Individuals with small isoforms (up to 22 KIV repeats) have 4–5 times higher Lp(a) concentrations compared to those who carry only large isoforms (more than 22 KIV repeats) [13, 15–18]. Depending on the population, about 30 to 70% of the variance in Lp(a) concentrations is explained by the apo(a) size polymorphism [19]. The causality of this relationship is most probably due to a more efficient maturation of smaller apo(a) proteins in the endoplasmatic reticulum [20, 21]. The entire *LPA* gene locus with all its variability explains up to 90% of Lp(a) variance [16, 22].

The apo(a) isoforms are important and major causal determinants of Lp(a) concentrations. Nevertheless, at an individual level same-sized isoforms may be associated with 200-fold different Lp(a) values [22, 23]. On the other hand, the inter-individual variation of alleles that are identical-by-descent within families is markedly smaller (only up to threefold) [23]. This implies that other genetic variants might influence Lp(a) concentrations in a substantial manner.

Since almost 15 years the investigation of single-nucleotide polymorphisms (SNPs) in the wider *LPA* gene region became of major importance. The two SNPs rs10455872 and rs3798220 revealed a pronounced association with high Lp(a) concentrations [24]. Both SNPs do not have any functional influence on the production or metabolism of Lp(a) and by that on the Lp(a) concentration. However, they “tag” small apo(a) isoforms which means that half of the small apo(a) isoform carriers carry at the same time at least one of the mutated alleles of the two SNPs [25]. Since they are easy to genotype in the laboratory on a large scale in many thousands of persons, they became an important tool to study the associations of the *LPA* gene with various disease conditions.

Besides these two SNPs genome-wide association studies in the wider *LPA* gene region identified more than 2000 SNPs to be genome-wide significantly associated with Lp(a) concentrations [26, 27]. The majority of them are functionally irrelevant or tag simply other SNPs that are influencing Lp(a) concentrations besides the K-IV repeat polymorphism as extensively reviewed recently [28••]. Important examples are the two splice site variants 4925G > A and 4733 G > A in the KIV type-2 repeat region. They are quite common with carrier frequencies of 22% and 38%, respectively and result in a pronounced decrease in Lp(a) concentrations of about 30 mg/dL and 13 mg/dL, respectively [29, 30•]. These two splice site variants represent the two most important genetic modifiers of Lp(a) concentrations besides the apo(a) isoform size [28••].

## The Basic Prerequisite for Treatment of High Lp(a) Is the Causality of Lp(a) for Cardiovascular Disease

The lack of a good animal model for Lp(a) research has limited investigations on the causality of Lp(a) with cardiovascular disease for a long time. However, the field was resurrected by genetics [19] by using the tools of Mendelian randomization studies which provided a very strong support of causality between Lp(a) concentrations and cardiovascular outcomes (discussed elsewhere [31]). The first time ever, the principle of a Mendelian randomization study was applied by Gerd Utermann’s group, even if that term was only coined 10 years later. After they had identified the apo(a) size polymorphism as an ideal genetic instrument in 1987 [13], they studied this polymorphism in several case–control studies of CHD patients and controls. They found that the small apo(a) isoforms were not only associated with higher Lp(a) concentrations but also with a higher frequency in patients with coronary heart disease [32, 33]. Many studies followed and a meta-analyses several years later revealed that carriers of small apo(a) isoforms had roughly a twice as high risk for cardiovascular events when compared to those who carried only large apo(a) isoforms [34]. The same finding was observed after the molecular characterization of the basis for the apo(a) size polymorphism had taken place and again those with a small number of K-IV repeats (coding for small apo(a) isoforms) had a higher risk [35]. Later very big studies from Denmark which investigated the sum of K-IV repeats of both Lp(a) alleles revealed similar results [12••, 36, 37]. Clarke and colleagues introduced for the first time the two SNPs, rs10455872 and rs3798220, and found that they were not only associated with high Lp(a) concentrations but also with a higher risk for coronary disease [24]. This has been confirmed by numerous studies and the two SNPs became two of the most intensively studied SNPs in the literature on cardiovascular disease. We recently used data from the UK Biobank in more than 400,000 individuals to support the causal relationship between Lp(a) and cardiovascular disease for the most recent EAS Lp(a) consensus statement on Lp(a) [9•, 38]. Those individuals who carried one of the two mutated alleles of the two SNPs had not only tenfold higher Lp(a) concentrations but a 47% higher risk to experience a cardiovascular event compared to those who carried only wildtype alleles of the two polymorphisms. Individuals who carried two of the mutated variants had almost 20-fold higher Lp(a) concentrations and a 89% higher risk for a cardiovascular event. Carriers of all these mentioned variants (small apo(a) isoforms determined by a low number of K-IV repeats, sum of K-IV repeats, certain SNPs) are exposed to a life-long exposure to higher Lp(a) concentrations and therefore a higher risk to develop a cardiovascular event (Fig. 2).

Conversely, rare genetic variants which result in loss of function [39, 40], or certain very common splice site variants [29, 30•] with pronounced Lp(a)-lowering effects, were found to be protective against the development of cardiovascular disease.

## What Can Currently Be Done in Case of High Lp(a) Concentrations?

There is a widespread misconception that a measurement of Lp(a) does not provide an advantage as long as no Lp(a)-lowering drug is on the market. However, this must be countered quite decisively, as it has been shown and argued in the latest EAS Lp(a) consensus statement [6••, 9•]. From the data provided in Fig. 1 based on this official statement, it can be concluded that (1) in case of high Lp(a) and a medium or large number of traditional risk factors, the overall global risk might be underestimated markedly and these risk factors have to be treated as good as possible to decrease the global risk of a given person, and (2) the treatment of these traditional risk factors should start as early as possible. The consensus statement provided examples for LDL cholesterol lowering: in case LDL cholesterol is elevated and treatment starts only at the age of 60 years instead of the age of 30 years, LDL-lowering has to be twice as much. Besides LDL cholesterol lowering, the normalization of an increased blood pressure, improved control of metabolic disturbances including diabetes mellitus, weight loss, and change in lifestyle (smoking cessation, increase of physical activity, a healthier dietary behavior, etc.) according to the various guidelines will contribute to a decrease of the global cardiovascular risk [6••, 9•]. This recommendation is supported by the population-based EPIC-Norfolk Study [41] that grouped participants at the baseline by seven modifiable risk factors (smoking status, high blood pressure, diabetes, cholesterol concentrations, body mass index, healthy diet, physical activity) and prospectively followed them for 11.5 years. When they analyzed the stratum of participants with Lp(a) concentrations above 50 mg/dL, they observed that those with a low and a medium number of these risk factors had only a third or two thirds of cardiovascular events, respectively, compared to those with a large number of these modifiable risk factors. This is even more compelling since all three groups had roughly the same medium Lp(a) concentrations (about 66 mg/dL). It is important to show these results to those healthcare professional colleagues who are until now neglecting Lp(a) as well as our patients and to tell them, that intervening on the modifiable risk factors is even more important in case of high Lp(a) concentrations as pointed out recently [42]. To simply wait for Lp(a)-lowering drugs without doing anything in the meanwhile is wrong for three main reasons: (1) after approval, these drugs will probably only be available in the secondary prevention setting until studies in the primary prevention setting are performed; (2) it is cynical and unethical to wait whether cardiovascular disease develops in a person with high Lp(a) since the first event is quite often fatal; and (3) it counteracts the move our society should make from “repair medicine” to a 4P Medicine approach (predictive, preventive, personalized, participatory) which focuses on prevention, health promotion, innovation, and awareness raising [43].

In some countries, Lp(a) apheresis is a possibility in patients with elevated Lp(a) (e.g., > 60 mg/dL) and progressive ASCVD (e.g., more than one ASCVD event) despite optimal treatment of all other risk factors. By this procedure Lp(a) levels are lowered by 60–70% per apheresis session with a rebound of Lp(a) levels that requires a weekly or bi-weekly treatment. Various studies suggest that regular apheresis may translate into clinical benefit [44–46], albeit large randomized, placebo-controlled trials are lacking (and would be hard to perform for ethical reasons). With this procedure not only Lp(a) is reduced but also other plasma lipoproteins (e.g., LDL cholesterol) and at the same time rheological parameters are improved.

## Specific Lp(a)-Lowering Drugs in Clinical Trials

As discussed recently [10, 47•], most specific Lp(a)-lowering therapies target the production of apo(a) in the liver cell by using RNA-targeting strategies. There are two possibilities: single-strand antisense oligonucleotide (ASO) or short interfering RNA (siRNA). Both are administered subcutaneously and are using Gal-NAc sugar chain facilitating direct and specific hepatic uptake through the ASGPR-1 receptor resulting in pronounced dose reduction.

The ASOs are 13–20 nucleic acid long and bind directly to the mRNA of apo(a) and form a complex with the intracellularly available RNAse H1 resulting in mRNA cleavage and thereby preventing the production of the targeted protein [48]. They act shorter and have therefore to be applied monthly. Compared to ASOs, double-stranded siRNAs enter the hepatocyte and are released from the endosome and the two RNA strands dissociate into the sense and antisense strand. The antisense strand forms a highly stable complex with the RNA-induced silencing complex (RISC) which induces the cleavage of the target mRNA, degradation by exonucleases and reduced synthesis of apo(a). Since the complex of siRNA with RISC is very stable, this results in a long-term cleavage of the targeted transcripts with a suppression of the protein production lasting more than 6 months [48].

Various clinical trials are ongoing. For example, Pelacarsen uses the ASO technology and results in an approximately 80% reduction in Lp(a) plasma levels with 60–80 mg subcutaneous dosing once every 4 weeks [49]. The phase III cardiovascular outcomes study will be finished probably by the end of 2025 (HORIZON, NCT 04023552). The siRNA technology is used by Olpasiran which reported a reduction of Lp(a) levels of up to more than 95% [50]. The recruitment for the cardiovascular outcomes study started and the study is expected to be finished by the end of 2026 (Ocean(a), NCT05581303). Zerlasiran (SNL360) is a further siRNA therapy, which resulted in the phase 1 study in a 98% reduction of Lp(a) concentrations following a single subcutaneous administration of 600 mg [51]. The phase II study is currently ongoing and is expected to be completed middle 2024 (NCT05537571). Finally, Lepodisiran is also using an siRNA approach and reported in a phase I study median dose-dependent decreases in Lp(a) concentrations > 90% for the 3 highest doses studied. The treatment effect lasted the longest in the highest dose of 608 mg and was still − 94% after 337 days of observation [52].

These mRNA-targeting therapies are highly effect therapies in terms of Lp(a)-lowering with only small side effects reported up to now. It has to be seen in the cardiovascular outcome trials, whether this pronounced Lp(a)-lowering translates in a lowering of the cardiovascular outcomes of interest.

A different approach is followed by Muvalaplin (LY3473329) which is an orally administered small molecule that inhibits Lp(a) formation. It binds to apo(a) KIV type 7 and KIV type 8 and thereby prevents the initial noncovalent interaction between apo(a) and apolipoprotein B100 of the LDL-particle. As a consequence, the disulfide bond between the two molecules is not built and the formation of Lp(a) is prevented. A phase I multiple ascending dose treatment evaluated the effect of taking daily doses of Muvalaplin (30 to 800 mg) or placebo for 14 days in patients with Lp(a) levels of 30 mg/dL or higher. The drug was tolerated well and resulted in a maximum placebo-adjusted Lp(a) reduction of 63 to 65%. Interestingly, similar effects were observed with daily doses of 100, 300, 500, and 800 mg [53]. A phase II, randomized, double-blind, placebo-controlled Study (KRAKEN) is currently running to investigate the efficacy and safety of oral once-daily administration of this drug in adults with elevated Lp(a) concentrations at high risk for cardiovascular events and is expected to be completed at the beginning of 2024 (NCT 05563246).

## Future Developments on Lp(a)-Lowering Strategies

Somatic gene-editing therapies shoot for long-lasting and highly likely permanent effects by editing the somatic DNA by introducing DNA changes. CRISPR/Cas9 system is one of the preferred technologies with a high efficiency as discussed by Stankov and Cuchel [54]. *PCSK9*, *ANGPTL3*, *LDLR*, and *APOC3* are current targets mostly in preclinical phase but *LPA* is already in the focus of some companies. Since this technology results in a permanent change of the somatic genome of an individual, long-term safety and ethical considerations are of high importance. In case these issues can be solved, a further interesting option might become available for persons with extremely high Lp(a) concentrations which will also circumvent compliance issues of oral lipid-lowering medications.

## Conclusions

Mendelian randomization studies during the early 1990s provided a strong support that Lp(a) is a causal risk factor for cardiovascular diseases. This knowledge paved the way for the development of specific Lp(a)-lowering therapies. Currently mRNA-targeting therapies are in clinical testing phase and result in a lowering of Lp(a) up to almost 100%. This modern therapies make Lp(a) concentration changes possible that one could never have imagined 20 years ago. It remains to be seen whether this results also in clinical benefits in terms of reduction of cardiovascular events.
